# Sex-dependent role for EPHB2 in brain development and autism-associated behavior

**DOI:** 10.1038/s41386-021-00986-8

**Published:** 2021-03-01

**Authors:** Ahlem Assali, Jennifer Y. Cho, Evgeny Tsvetkov, Abha R. Gupta, Christopher W. Cowan

**Affiliations:** 1 Department of Neuroscience, Medical University of South Carolina, Charleston, SC USA; 2 Medical Scientist Training Program, Medical University of South Carolina, Charleston, SC USA; 3 Departments of Pediatrics, Child Study Center, and Neuroscience, Yale School of Medicine, New Haven, CT USA

**Keywords:** Autism spectrum disorders, Development of the nervous system

## Abstract

Autism spectrum disorder (ASD) is characterized by impairments in social communication and interaction and restricted, repetitive behaviors. It is frequently associated with comorbidities, such as attention-deficit hyperactivity disorder, altered sensory sensitivity, and intellectual disability. A de novo nonsense mutation in *EPHB2* (Q857X) was discovered in a female patient with ASD [13], revealing *EPHB2* as a candidate ASD risk gene. EPHB2 is a receptor tyrosine kinase implicated in axon guidance, synaptogenesis, and synaptic plasticity, positioning it as a plausible contributor to the pathophysiology of ASD and related disorders. In this study, we show that the Q857X mutation produced a truncated protein lacking forward signaling and that global disruption of one *EphB2* allele (*EphB2*^*+/−*^) in mice produced several behavioral phenotypes reminiscent of ASD and common associated symptoms. *EphB2*^*+/−*^ female, but not male, mice displayed increased repetitive behavior, motor hyperactivity, and learning and memory deficits, revealing sex-specific effects of EPHB2 hypofunction. Moreover, we observed a significant increase in the intrinsic excitability, but not excitatory/inhibitory ratio, of motor cortex layer V pyramidal neurons in *EphB2*^*+/−*^ female, but not male, mice, suggesting a possible mechanism by which EPHB2 hypofunction may contribute to sex-specific motor-related phenotypes. Together, our findings suggest that EPHB2 hypofunction, particularly in females, is sufficient to produce ASD-associated behaviors and altered cortical functions in mice.

## Introduction

Autism spectrum disorder (ASD) is a neurodevelopmental disorder (NDD) characterized by impairments in social communication and interaction, and restricted, repetitive patterns of behavior, interests, or activities [1]. About 1 in 54 children are diagnosed with ASD in the United States [2], but the pathophysiology of ASD remains unclear. Decades of research indicate that a majority of ASD risk is genetic, but complex interactions with environmental factors likely play a role [3]. Moreover, ASD is more prevalent in males than females, but the sex-specific mechanisms underlying ASD risk remain elusive. De novo and rare, inherited copy number variants and single nucleotide variants (SNVs) affecting hundreds of genes have been associated with ASD [4]. This genetic heterogeneity likely contributes to the well-described variation in ASD symptom severity and the presence or absence of common comorbidities, like intellectual disability (ID), attention-deficit hyperactivity disorder (ADHD), anxiety disorders, sleep disruptions, changes in sensitivity to sensory stimuli, and gastrointestinal problems [5]. However, testing whether and how a candidate ASD risk gene produces ASD-associated symptoms remains a major challenge in human patients. One approach involves the generation of mutant mice bearing a heterozygous ASD-linked variant and testing whether that variant produces ASD-related behaviors and altered brain functions. Linking genotype to phenotype could provide key insights into the role of a specific gene in ASD pathophysiology and a path toward developing personalized treatments for affected individuals.

Convergent findings from clinical neuroimaging and human postmortem brain analyses suggest that the pathophysiology of ASD is linked to widespread disruptions in neuronal connectivity in the developing brain [6]. Several genetic studies have linked ASD to axon guidance dysregulation and synaptic dysfunction, supporting the idea that ASD is a synaptopathy [7]. Mutations in synapse-related genes, such as *DLG-4*, *FMR1*, and members of the *SHANK, NEUREXIN* and *NEUROLIGIN* families, have been identified in ASD individuals, and mice carrying loss-of-function mutations in these genes often display ASD-related behaviors [7], such as alterations in social and repetitive behaviors. The axon guidance- and synapse-related gene, *EPHB2*, has emerged as a potential risk gene for multiple neuropsychiatric disorders, including schizophrenia, depression, and anxiety disorders [8–12]. In addition, *EPHB2* emerged as a candidate ASD risk gene after a de novo nonsense mutation was discovered in a female patient with ASD in a whole-exome sequencing study of ASD families from the Simons Simplex Collection (SSC) [13]. EPHB2 is a tyrosine kinase receptor activated upon binding to clusters of ephrin-B ligands, and the binding of ephrin-B to EphB produces bidirectional signaling important for axon guidance, synapse formation, and plasticity during development and in adulthood (reviewed in [14]). EPHB2 forward signaling requires autophosphorylation at two juxtamembrane tyrosine residues (Y596 and Y602) located within the intracellular domain, which, in turn, mediates the recruitment of SH2 domain-containing adapter and signaling proteins involved in cytoskeletal remodeling, cell adhesion, and synapse development [15–17].

In this study, we examined the de novo Q857X ASD-associated nonsense mutation and several rare, inherited missense variants in *EPHB2*. The ASD-linked de novo mutation produced a truncated EPHB2 protein lacking forward signaling. In contrast, the 19 rare missense variants, similarly distributed in both ASD and neurotypical individuals, produced no obvious defects in protein stability or forward signaling capability. Since we hypothesized that the *EPHB2* Q857X mutation contributes to the female patient’s ASD phenotype, we examined the effects of global disruption of one *EphB2* allele (*EphB2*^*+/−*^) in both male and female mice to assess the potential of EPHB2 hypofunction for producing ASD-associated behaviors and/or altered cortical neuron function. Our findings revealed that EPHB2 hypofunction in mice produced several female-specific ASD-associated behaviors, including increased repetitive behavior, motor hyperactivity, and learning and memory deficits. We also detected an increase in cortical layer V motor neuron intrinsic excitability, specifically in *EphB2*^*+/−*^ females, but no clear changes of excitatory or inhibitory synaptic transmission. Together, our findings suggest that heterozygous, loss-of-function mutations in *EphB2* are sufficient in females, but not males, to produce ASD-associated symptoms and altered cortical function.

## Materials and methods

### Animals

*EphB2*^*+/−*^ mice, previously named *Nuk*^*1/+*^ mice [18], were maintained on a C57BL/6J background and group housed (2–5 mice/cage) with same-sex littermates on a 12 h light–dark cycle with constant access to food and water. *EphB2*^*+/−*^ mice were extensively backcrossed (>10 generations) to congenicity on C57BL/6, and all crosses for behavior experiments were performed with a C57BL/6 parent from Jackson Labs. All the behavior and electrophysiology experiments were performed using 2–4 month-old male and female mice, except for pup ultrasonic vocalization (USV) recordings, performed at postnatal days 5/6 and 10. All procedures were conducted in accordance with the MUSC Institutional Animal Care and Use Committee and NIH guidelines.

### Site-directed mutagenesis

The 21 different variants in *EPHB2* were generated via site-directed mutagenesis (Supplementary Fig. 1). *HEK293T cell transfection*. To overexpress EPHB2 mutant proteins, HEK293T cells were transfected with pCDNA3 plasmids expressing the different *EPHB2* variants using the calcium phosphate transfection method. *Western blot*. Membranes were incubated in primary antibodies (rabbit anti-HA, 1:1000, Sigma #H6908; rabbit anti-phospho EPH [19], 1:1000), and in secondary antibodies (LI-COR #926-32211; Goat anti-rabbit, 1:20000).

### Behaviors

*Social interaction*. Mice were allowed to explore a three-arena apparatus (Stoelting #60450) for 10 min. Mice were then removed, while a non-familiar conspecific mouse (same age and sex) and a novel object were placed in holding chambers in the side arenas. An interaction zone (circle with a 16.5 cm diameter) was defined around each holding chamber. Experimental mice were returned to the center arena and recorded for 10 min using ANY-maze behavior tracking software (Stoelting) to measure the time spent interacting with the novel mouse and novel object. Data are reported as time spent in each interaction zone. *Pup ultrasonic vocalization (USV)*. USVs were recorded from isolated pups at P5/6 and P10 for 3 min immediately following separation from their mother and littermates, in a sound-attenuated chamber, using Avisoft UltraSoundGate equipment (UltraSoundGate 116Hb with Condenser Microphone CM16; Avisoft Bioacoustics, Germany). USVs were quantified using Avisoft SASLab Pro (Avisoft Bioacoustics, Germany). Data are reported as number of USVs generated during the 3 min of recording. *Elevated plus maze*. Mice were placed in the center of an elevated plus maze that has two open arms and two closed arms (Stoelting #60140) in white light (100 lux) and recorded for 5 min using ANY-maze behavior tracking software (Stoelting). Data are reported as time spent in open arms. *Open field*. Mice were placed in a white open field box (44 cm^2^) in white light (130 lux) and recorded for 5 min using ANY-maze behavior tracking software (Stoelting). Data are reported as time spent in a predefined zone (14 × 14 cm) in the well-lit center of the box. *Locomotor activity*. Mice were placed in the dark for 1 h in an Open Field Activity, Infrared Photobeam Activity Test Chamber (Med Associates), that has two arrays of photobeams to measure vertical (jumping, rearing) and horizontal locomotor movements. Data are reported as total distance traveled, jump time, and vertical and horizontal repetitive fine movement time. *Fear conditioning (FC)*. After 2 min of acclimation in a FC chamber (Med Associates), mice were exposed three times (once/minute) to an auditory tone (30 s; 90 dB) followed each time by a mild footshock (0.5 mA). Freezing behavior was recorded using a video tracking system (Video Freeze V2.6; Med Associates). Twenty-four hours later, the freezing behavior of mice was recorded when the mice returned to the chamber (Test—context) for 6 min, and when the mice were reexposed to the auditory tone in a new context for another 6 min (3 min without tone (Test—altered context) followed by 3 min during which the tone was played (Test—cue/altered context)). Data are reported as percent of time the mouse is immobile. *Startle response to shock, acoustic startle response, and prepulse inhibition (PPI)*. To measure startle response to shock and acoustic startle response, mice were exposed to footshocks delivered by S/A Aversive Stimulators (Med Associates #ENV-414S) connected to wire grid floors of the animal holder, or to white-noise pulses at different intensities. Startle responses were measured using the Startle Reflex System and Advanced Startle software program (Med Associates).

### Myelin staining

Sections were stained for myelin using the BrainStain Imaging kit (ThermoFisher #B34650; FluoroMyelin, 1:300), following the kit protocol. Cortical thickness, corpus callosum (CC) thickness, and dorsal striatal area were measured using ImageJ software.

### Electrophysiology

All acute-slice electrophysiological experiments were performed in *EphB2*^*+/−*^ mice and their wild-type (WT) littermates.

### RT-qPCR

RNA extraction was performed using the miRNeasy Mini kit (Qiagen #1038703), following the kit’s protocol. Total RNA was reverse-transcribed using Superscript III (Invitrogen) with random hexamers, following the kit’s protocol. Quantitative real-time PCR was performed using the CFX96 qPCR instrument (Bio-Rad), the iTaq Universal SYBR Green Supermix (Bio-Rad), and primers specific to *EphB2* (forward: 5′CAACGGTGTGATCCTGGACTAC3′, reverse: 5′CACCTGGAAGACATAGATGGCG3′ used for Supplementary Fig. A and B; forward: 5′GATGGTACATCCCCCATCAG3′, reverse: 5′GCCAGTTGTTCTGGCTTGAC3′ used for Supplementary Fig. C and D). *Gapdh* was used to normalize gene expression in each sample. The data are reported as delta delta Ct.

### Statistical analysis

Two-way ANOVAs and three-way ANOVAs, with Sidak’s post hoc comparison, and unpaired *t*-tests were performed for statistical analyses, using GraphPad Prism software. ^#^*p* < 0.1, *p < 0.05, **p < 0.005, ***p < 0.0005, ns: not significant. All *p* values are reported in Supplementary Tables 1–3. 

Additional materials and methods details are found in Supplementary materials and methods.

## Results

### Detection of rare, nonsynonymous single nucleotide variants in *EPHB2* gene

**Fig. 1:**
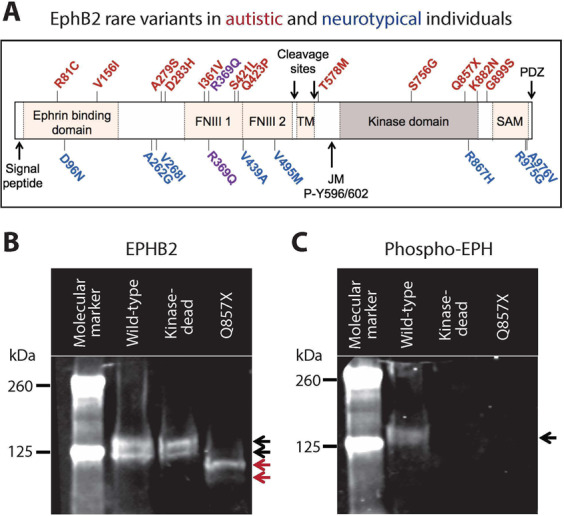
The de novo mutation Q857X induces EPHB2 truncation and loss of autophosphorylation activity. **A** Scheme representing EPHB2 protein, with EPHB2 domains and EPHB2 rare variants in autistic individuals (red), in neurotypical individuals (blue), and in both (purple). Western blots of HA-EPHB2 (**B**) and of Y602 phospho EPH (**C**), for wild-type EPHB2 (black arrows), kinase-dead EPHB2 (point mutation in EPHB2 kinase domain that inhibits kinase activity), and Q857X EPHB2 (red arrows). FNIII 1 fibronectin type-III 1, FNIII 2 fibronectin type-III 2, TM trans-membrane, JM P-Y596-602 juxtamembrane phosphorylation site at tyrosine 596 and 602, SAM sterile alpha motif domain, PDZ PSD-95/Dlg/ZO-1 domain.

The identification of the de novo nonsense mutation (Q857X) revealed *EPHB2* as a candidate risk gene for ASD [13]. To explore this possibility further, we examined the results of a separate project (unpublished data) involving PCR amplification and Sanger sequencing across all *EPHB2* coding exons in 864 ASD probands and both biological parents from the SSC [20] and 831 unrelated individuals from the National Institute of Neurological Disorders and Stroke (NINDS) Neurologically Normal Caucasian Control Panel (coriell.org/1/NINDS). Resequencing analysis confirmed the presence of a de novo *EPHB2* Q857X mutation and also identified 11 rare (<1% frequency as per the racially diverse gnomAD v2 database, that includes males and females, gnomad.broadinstitute.org) missense SNVs in the ASD probands (Table 1 and Fig. 1A). Nine rare *EPHB2* SNVs were found in the neurotypical controls (Table 1 and Fig. 1A), and one of these rare variants was found in both ASD and neurotypical individuals (R369Q, Table 1, bold letters, and Fig. 1A, purple letters). All but two of the Sanger sequencing-confirmed rare variants were heterozygous and were found in one of the ASD proband’s neurotypical parents, indicating that the ASD-associated, rare missense variants were inherited. Interestingly, the ASD proband rare variants all coded for amino acid changes spanning the entire length of the EPHB2 protein, including amino acid changes within the critical tyrosine kinase domain (amino acids 621–884) (Table 1 and Fig. 1A). None of the rare SNVs that are unique to the ASD probands are sufficient to cause ASD since an unaffected parent possesses the same genetic variant. However, we hypothesized that unique proband SNVs might confer increased genetic risk for developing ASD if the SNVs were disproportionately deleterious to EPHB2 function. In heterologous cell studies, we failed to detect any obvious changes in the EPHB2 mutant protein’s expression, molecular weight, or autophosphorylation of its juxtamembrane tyrosines (Table 1 and Supplementary Fig. 2). In addition, the de novo G899S *EPHB2* mutation [21] did not induce any changes to the EPHB2 protein (Table 1 and Supplementary Fig. 2).

**Table 1:** *EPHB2* rare variants in autistic and neurotypical individuals.

Variant	Inherited/de novo	Number of cases	Het/Hom	Exon	Protein domain	Protein	P-Y602
*EPHB2* rare variants in SSC probands (*n* = 864)							
R81C	Inherited	1	Het	3	Ephrin binding domain	Normal	✔
V156I	Inherited	1	Het	3	Ephrin binding domain	Normal	✔
A279S	Inherited	1	Het	4	Extracellular	Normal	✔
D283H	Inherited	2	Het	4	Extracellular	Normal	✔
I361V	Inherited	3	Het	5	FNIII 1	Normal	✔
R369Q	Inherited	4	Het	5	FNIII 1	Normal	✔
S421L	Inherited	1	Het	5	FNIII 1	Normal	✔
Q423P	Inherited	1	Het	5	FNIII 1	Normal	✔
T578M	Inherited	1	Het	9	Intracellular	Normal	✔
S756G	Inherited	1	Het	12	Kinase domain	Normal	✔
Q857X	De novo	1	Het	14	Kinase domain	Truncated	X
K882N	Inherited	1	Het	14	Kinase domain	Normal	✔
G899S	De novo	1	Het	14	Intracellular	Normal	✔
*EPHB2* rare variants in NINDS neurotypical controls (*n* = 831)							
D96N	Unknown	1	Het	3	Ephrin binding domain	Normal	✔
A262G	Unknown	1	Het	3	Extracellular	Normal	✔
V268I	Unknown	1	Het	3	Extracellular	Normal	✔
R369Q	Unknown	3	Het	5	FNIII 1	Normal	✔
V439A	Unknown	1	Het	6	FNIII 2	Normal	✔
V495M	Unknown	1	Het	7	FNIII 2	Normal	✔
R867H	Unknown	1	Het	14	Kinase domain	Normal	✔
R975G	Unknown	1	Het	16	SAM domain	Normal	✔
A976V	Unknown	1	Het	16	SAM domain	Normal	✔

In contrast to the inherited variants, the de novo *EPHB2* Q857X mutation produced a truncated EPHB2 protein (Fig. 1B, red arrows). The *EPHB2* WT and the de novo *EPHB2* Q857X mutation both produced proteins with two molecular weights, possibly reflecting a proteolytic cleavage (Fig. 1B, black and red arrows). Moreover, the *EPHB2* Q857X mutant lacked autophosphorylation of the juxtamembrane tyrosines (Fig. 1C), consistent with the predicted truncation of the EPHB2 tyrosine kinase domain. This finding confirms the original presumption that the de novo *EPHB2* Q857X mutation produces a severe, deleterious effect on EPHB2 protein function [13].

### *EphB2*^*+/−*^ female mice display several ASD core and common associated behaviors

**Fig. 2:**
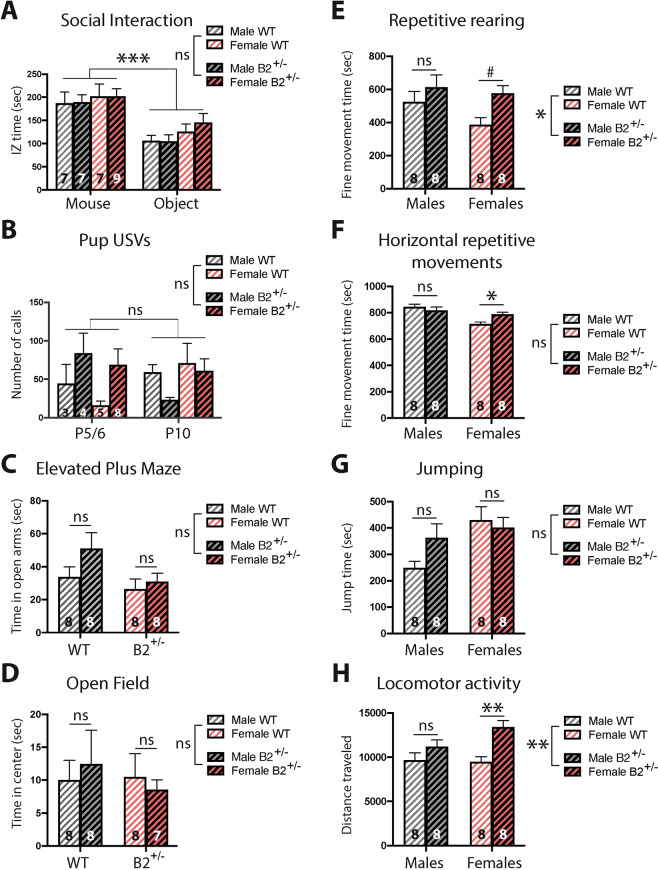
*EphB2*^*+/−*^ females, but not males, display repetitive behaviors and hyperactivity. **A** Both WT and *EphB2*^*+/−*^ male and female mice show a significant preference for a novel mouse over a novel object in a 3-arena social interaction assay, with no genotype differences (three-way ANOVA; main effect of IZ: *p* = 0.0002). **B** *EphB2*^*+/−*^ male and female pups show no difference in the number of USVs at P5/6 or P10 compared to their WT littermates during maternal separation (three-way ANOVA). *EphB2*^*+/−*^ male and female mice show normal anxiety-like behavior (two-way ANOVA) in the elevated plus maze (**C**) and open field (**D**). **E** *EphB2*^*+/−*^ mice show an increase in repetitive rearing (two-way ANOVA; main effect of genotype; *p* = 0.0234), post hoc analysis shows a statistical trend to an increase in *EphB2*^*+/−*^ females, but not in males (post hoc analysis: for females, *p* = 0.054, and for males, *p* = 0.4986). **F** *EphB2*^*+/−*^ females, but not males, show repetitive horizontal movements (two-way ANOVA; no main effect of genotype but significant interaction: *p* = 0.0251, post hoc analysis: for females, *p* = 0.0385, and for males, *p* = 0.6315). **G** No difference in repetitive jumping between *EphB2*^*+/−*^ and WT mice (two-way ANOVA). **H** *EphB2*^*+/−*^ mice are hyperactive (two-way ANOVA; main effect of genotype: *p* = 0.0011), post hoc analysis shows a significant hyperactivity in *EphB2*^*+/−*^ females, but not in males (post hoc analysis: for females, *p* = 0.0018, and for males, *p* = 0.3034). WT wild type, IZ interaction zone, USVs ultrasonic vocalizations. Data are represented as mean ± SEM. Statistical significance was determined by two-way ANOVA or three-way ANOVA. ^#^*p* < 0.1, *p < 0.05, **p < 0.005, ***p < 0.0005, ns not significant. The number of animals is indicated within each bar for each experiment.

To test whether a heterozygous, deleterious mutation of *EphB2* in mice is sufficient to produce behavioral phenotypes reminiscent of the core symptoms of ASD or other common ASD-associated symptoms, we examined some relevant behaviors in heterozygous, loss-of-function *EphB2* mutant mice (*EphB2*^*+/−*^) and WT littermate controls. Global *EphB2*^*+/−*^ mice appeared normal physically, and they were born at the expected Mendelian frequency. Compared to WT littermates, the *EphB2*^*+/−*^ males and females showed similar social preference (Fig. 2A) and number of USVs, a putative species-appropriate form of oral communication, following maternal separation (Fig. 2B). To examine restricted, repetitive interests or activities in the mice, we analyzed repetitive horizontal and vertical stereotypic activity. *EphB2*^*+/−*^ female, but not male, mice showed a significant increase in horizontal fine motor movements, often interpreted as enhanced stereotypic behavior, with no difference in repetitive rearing (Fig. 2E, F). Moreover, *EphB2*^*+/−*^ female, but not male, mice exhibited a significant increase in locomotor activity in a novel environment, often interpreted as motor hyperactivity (Fig. 2H), with no differences in jumping (Fig. 2G). As shown in Fig. 2H (and Supplementary Fig. 5), we detected no significant sex differences in locomotor activity in the WT, C57BL/6 congenic mice. Together, these data reveal a sex-specific role for EPHB2 in regulating hyperactivity and repetitive motor behaviors.

**Fig. 3:**
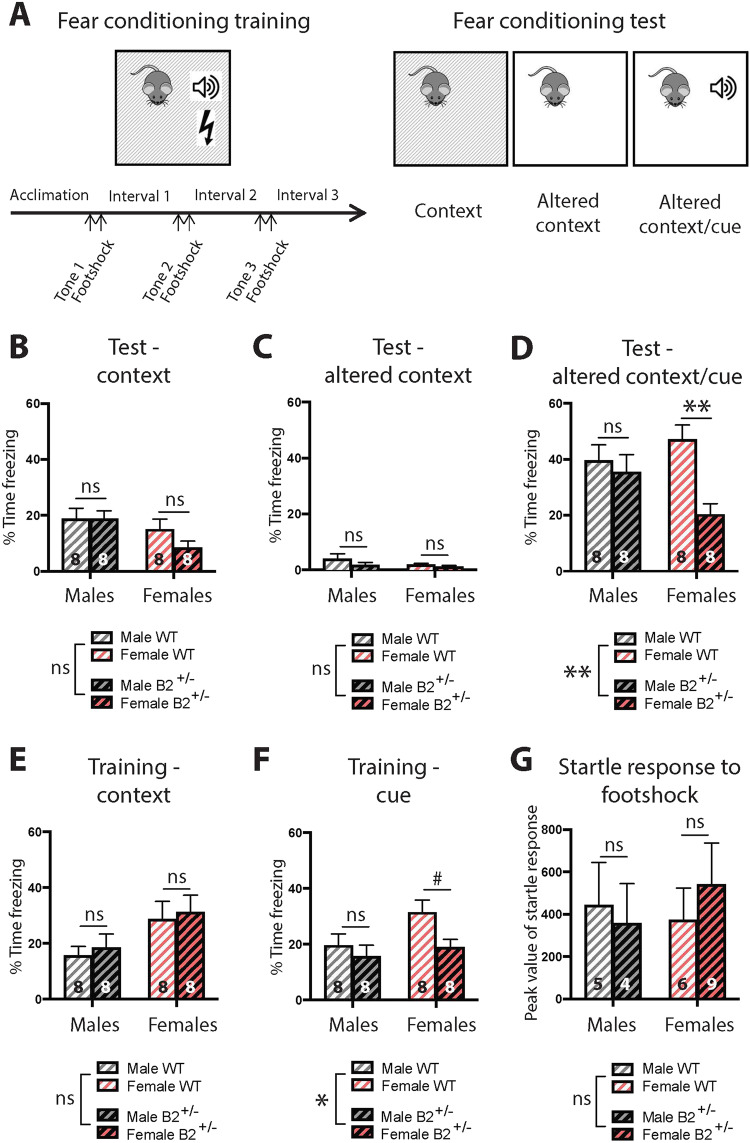
*EphB2*^*+/−*^ females, but not males, display deficits in cue-induced fear memory. **A** Scheme representing the fear conditioning (FC) assay. During FC training, the animals are exposed to three footshocks (0.5 mA) preceded by an auditory tone (cue) in a specific chamber (context). Twenty-four hours later, during FC test, the animals are first placed in the same context, then placed in an altered context, and finally reexposed to the cue in the altered context. **B** *EphB2*^*+/−*^ males and females show normal context-induced fear memory (two-way ANOVA). **C** No difference in freezing time during the FC test in an altered context (two-way ANOVA). **D** *EphB2*^*+/−*^ females, but not males, display a robust decrease in freezing time when reexposed to the cue in an altered context (two-way ANOVA; significant interaction: *p* = 0.042; main effect of genotype: *p* = 0.007; post hoc analysis: for females, *p* = 0.0026, and for males, *p* = 0.8275). **E** No difference in freezing time during the last interval following the last footshock of fear conditioning training in the chamber between *EphB2*^*+/−*^ males or females and WT mice (two-way ANOVA). **F** *EphB2*^*+/−*^ females, but not males, present a statistical trend to decreased cue-induced freezing time during the last auditory tone of FC training, preceding the last footshock (two-way ANOVA; main effect of genotype: *p* = 0.0437; post hoc analysis: for females, *p* = 0.0616, and for males, *p* = 0.7272). **G** No difference in startle response to footshocks at 0.5 mA between *EphB2*^*+/−*^ and WT males and females (two-way ANOVA). WT wild type. Data are represented as mean ± SEM. Statistical significance was determined by two-way ANOVA with Sidak’s post hoc comparison. ^#^*p* < 0.1, *p < 0.05, **p < 0.005, ns not significant. The number of animals is indicated in each bar for each experiment.

Next, we examined anxiety-like behavior and deficits in learning and memory, since these are common comorbidities observed in individuals with ASD. No significant differences were detected between *EphB2*^*+/−*^ and WT mice in two different anxiety-like behavior tests (Fig. 2C, D and Supplementary Fig. 5). However, in the classical FC assay (Fig. 3A), where mice learn to associate a discreet cue (auditory tone) with the presentation of aversive mild footshocks (0.5 mA), we found that *EphB2*^*+/−*^ females, but not males, showed a robust deficit in cue-induced freezing behavior (Fig. 3D), but not a significant deficit in context-associated freezing (Fig. 3B) or auditory cue-induced freezing during training (Fig. 3F). The female *EphB2*^*+/−*^*-*specific deficit in fear memory was not caused by differences in aversive shock sensitivity, as the shock-induced startle response at 0.5 mA was not different by sex or genotype (Fig. 3G). Taken together, our findings reveal a sex-dependent role for *EphB2* in cue-induced fear/threat memory.

Another common associated symptom of ASD is altered sensitivity to sensory stimuli. To examine sensitivity to auditory stimuli, we measured startle response to different intensities of a white-noise burst. Neither *EphB2*^*+/−*^ female or male mice showed a significant change in acoustic startle response (Supplementary Fig. 3A, B). We also assessed sensorimotor gating using PPI of startle—a neurological process reflecting information processing of sensory, motor, and cognitive information. PPI is reduced in multiple neuropsychiatric disorders, including schizophrenia, ASD, obsessive-compulsive disorder, bipolar disorder, and many others [22]. Compared to their WT littermates, *EphB2*^*+/−*^ mice displayed a significant decrease in the magnitude of PPI of startle (Supplementary Fig. 3C). These findings suggest a sex-independent role for EPHB2 in information processing.

### EphB2 hypofunction increases intrinsic excitability of motor cortex layer V pyramidal neurons

**Fig. 4:**
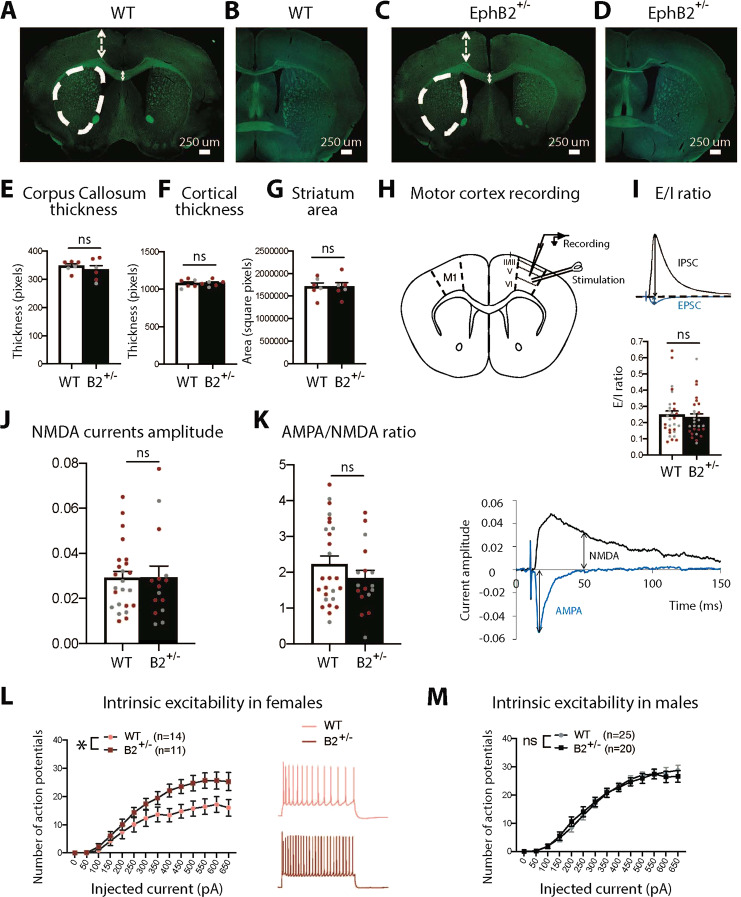
*EphB2*^*+/−*^ females, but not males, display an increased intrinsic excitability in layer V motor cortex (M1) pyramidal neurons. **A**–**D** A myelin stain shows normal corpus callosum and anterior commissure structure in *EphB2*^*+/−*^ compared to WT mice. No difference in corpus callosum thickness (**E**), in cortical thickness (**F**), and in striatal area (**G**) between *EphB2*^*+/−*^ and WT males and females (*t*-tests). **H** Scheme representing acute, ex vivo coronal slice electrophysiological recording in M1 (Bregma ~0.65 mm). **I** No difference in the excitation/inhibition balance (E/I) in *EphB2*^*+/−*^ males or females compared to WT mice (*t*-test). The traces represent the excitatory postsynaptic current (EPSC) and the inhibitory postsynaptic current (IPSC). **J** *EphB2*^*+/−*^ females and males show normal NMDA receptor-mediated currents amplitude and normal AMPA/NMDA ratio (*t*-tests). The traces represent the NMDA and AMPA receptor-mediated currents. **E**–**G**, **I**–**K** Color-coded dots on the graphs show the data for males (gray) and females (red). Two-way ANOVAs showed no sex differences. **L**, **M** *EphB2*^*+/−*^ females present an increase in the number of action potentials compared to WT females (L; two-way ANOVA; main effect of genotype: *p* = 0.0334). The traces represent the action potentials in *EphB2*^*+/−*^ and WT females. No difference in intrinsic excitability between *EphB2*^*+/−*^ and WT males (M; two-way ANOVA). The number of cells is indicated for each experiment. Data are represented as mean ± SEM. *p < 0.05, ns not significant.

Human and rodent studies indicate that the cortico-striatal circuit is critical for motor activity and repetitive behaviors, and altered function of this circuit is associated with ASD and other neuropsychiatric disorders [23]. Since *EphB2*^*+/−*^ females exhibited motor hyperactivity and repetitive behaviors, we examined motor cortex layer V pyramidal neuron physiology in WT and *EphB2*^*+/−*^ mice. We detected no changes by genotype or sex (Fig. 4A–G) in cortical thickness, striatum area, or general appearance or thickness of the CC and posterior branch of the anterior commissure (pAC) (Fig. 4A–D, E), which are both abnormal in *EphB*^*−/−*^ mice [24, 25]. To examine excitatory and inhibitory synaptic transmission and intrinsic excitability in the M1 motor cortex, we performed patch-clamp recordings from acute, ex vivo coronal slices from young adult WT or *EphB2*^*+/−*^ mice (Fig. 4H). Using voltage-clamp conditions, we examined both the electrically evoked AMPA- and GABA-A-mediated currents in the same layer V pyramidal cell. However, we observed no differences in the excitatory/inhibitory (E/I) ratio by sex or genotype (Fig. 4I). Moreover, despite the well-described regulation of NMDA receptors function by EPHB2, we detected no significant differences in *EphB2*^*+/−*^ mice in NMDA receptor-mediated current amplitude or AMPA/NMDA ratio (Fig. 4J, K). In contrast, using current-clamp mode, we observed a significant increase in intrinsic excitability in *EphB2*^*+/−*^ females compared to WT females, but not in males (Fig. 4L, M), suggesting that EPHB2 hypofunction produces a sex-specific enhancement of excitability in cortical M1 layer V projection neurons.

## Discussion

The discovery of a de novo nonsense mutation (Q857X) in *EPHB2* in a female patient with ASD, predicted to be severely functionally damaging, revealed *EPHB2* as a candidate ASD risk gene [13]. We have identified 19 additional rare missense variants in *EPHB2* in autistic and neurotypical individuals. However, none of the rare variants, observed in both ASD individuals and neurotypical controls, produced a detectable change in EPHB2 protein expression, molecular weight, or autophosphorylation at critical juxtamembrane tyrosines required for forward signaling. This suggests that none of these inherited rare variants are severely damaging to EPHB2 forward signaling. In contrast, the ASD-associated *EPHB2* Q857X mutation induced a large protein truncation, as expected, and it eliminated detectable autophosphorylation of juxtamembrane tyrosines, which is critical for normal EPHB2 function. Interestingly, *EphB2*^*+/−*^ females, but not males, displayed repetitive behavior, a core symptom of autism, as well as autism-associated symptoms, such as motor hyperactivity and learning and memory deficits. *EphB2*^*+/−*^ females also showed an increase in intrinsic excitability of motor cortex layer V pyramidal neurons. Taken together, our findings revealed that EPHB2 hypofunction selectively in females produced several ASD-associated behaviors and altered cortical neuron function.

Rare variants contribute to ASD etiology in humans [26]. Since female mice were more impacted by *EphB2* hypofunction, we assessed whether *EPHB2* rare, inherited variants were enriched in females with ASD. However, 80% of *EPHB2* rare, inherited variants were detected in male probands, which is proportional to the 4:1 ratio of males to females in the general ASD population. Similarly, within the neurotypical population, we observed that 45% of *EPHB2* rare, inherited variants were found in males, which is similar to the ratio of males to females in the general population. In addition, while our examination of all EPHB2 protein functions was not exhaustive, and we cannot exclude the possibility that some rare, inherited variants could alter ephrin binding affinity, subcellular localization, and more, the observation that there are a similar number of rare variants in ASD cases and neurotypical controls (Table 1) and that these missense variants in ASD cases are all inherited from an unaffected parent suggests that they are unlikely to be severely damaging to EPHB2 function and unlikely to confer significant risk for ASD by themselves. However, it remains possible that *EPHB2* rare variants, in combination with other ASD risk genes or environmental factors, could contribute to risk for one or more neurodevelopment disorders. It is also important to note that genetic manipulation in the *EphB2*^*+/−*^ mice produces a true loss-of-function *EphB2* allele, whereas the human *EPHB2* Q857X de novo mutation produces a truncated protein lacking kinase activity and some C-terminal domains. As such, the *EPHB2* Q857X mutation might produce ASD and autism-associated symptoms through a dominant interfering or gain-of-function effect during neurotypical development, and future studies with *EphB2* Q857X knock-in mice will be important to assess that possibility.

Why and how EPHB2 hypofunction produces repetitive and hyperactive motor behaviors and learning and memory deficits, specifically in females, remains unclear. Female sex hormones play important roles in brain development and synapse function [27], and EPHB2 regulates synapse development and plasticity [14], but future studies will be required to determine the precise reasons for the interaction between EPHB2 hypofunction and sex. We considered the possibility that EPHB2 might be expressed in the female brain at lower levels than males, thus creating a sex-specific gene dosage hypersensitivity; however, in WT and *EphB2*^*+/−*^ mice, overall *EphB2* expression in the mouse brain was not different between males and females at either postnatal days 2–6 or in adults (Supplementary Fig. 4). Follow-up studies with greater power to detect potential small differences in males will be required to determine whether certain effects of EPHB2 hypofunction are always specifically observed in females, or can be detected in males with a smaller amplitude than in females. In the future, understanding the sex-specific mechanisms of EPHB2 function in brain development and behavior will be important, particularly since the one example of a de novo functionally damaging mutation in *EPHB2* (Q857X) was observed in a female with ASD symptoms.

The PPI deficit in the *EphB2*^*+/−*^ mice suggests abnormalities in sensorimotor gating, and PPI deficits are observed in multiple neuropsychiatric disorders, including schizophrenia. Of note, several recent human genome-wide association studies identified *EPHB2* as a possible risk gene for schizophrenia [10, 12]. In addition, individuals with Fragile X Syndrome, the most common genetic cause of ASD, also display PPI deficits [28].

Prior studies have shown that complete loss of EPHB2 (*EphB2*^*−/−*^) or EPHB2 forward signaling (*EphB2*^*lacZ/lacZ*^) causes deficits in classical FC [29–31]. In addition, EPHB2 regulates synaptic connectivity and plasticity in both the hippocampus and amygdala [11, 32–37], which are key brain structures involved in context- and cue-associated FC. Our data show that the loss of a single allele of *EphB2* in females, but not males, is sufficient to produce deficits in cue-, but not context-associated, FC, suggesting a possible role for sex hormones in regulating EPHB2 function in auditory-cued fear memory circuits, like the basolateral amygdala. This idea is consistent with the observation that activation of a photoactivable form of EPHB2 in the amygdala enhances auditory fear memory through a forward signaling role [31].

Since EPHB2 plays a noted role in axon guidance during development [14], the behavior deficits in *EphB2*^*+/−*^ females might be caused, at least in part, by subtle axon guidance defects. EPHB2 is required for the proper formation of several major axon tracts, including the pAC and the CC [24, 25], and these structures are abnormal in a subset of individuals with ASD [38]. Moreover, EPHB2 cooperates with EPHB1, a closely related guidance receptor, to regulate the proper navigation of a subpopulation of sensory cortical axon projections [24]; altered sensory sensitivity is observed frequently in individuals with an ASD diagnosis [39]. However, the loss of function of a single *EphB2* allele in female or male mice did not produce any of the previously described axon guidance deficits detected in *EphB2*^−/−^ mice [24, 25].

EPHB2 can regulate synaptogenesis and AMPA and NMDA receptor functions in developing pyramidal neurons [14]. Since multiple motor behaviors were enhanced in female *EphB2*^*+/−*^ mice, we chose to initially examine synaptic function of pyramidal neurons in the motor cortex of male and female *EphB2*^*+/−*^ mice and littermate controls. While we failed to detect changes in AMPA, NMDA, or GABA-A receptor-mediated currents (Fig. 4), we did observe a female-specific significant increase in intrinsic excitability, suggesting that EPHB2 might regulate, directly or indirectly, potassium (K+) channel functions. This finding is particularly interesting since human brain imaging studies of ASD individuals [23] show altered cortico-striatal systems. Similarly, in some mouse models of ASD, such as the *Fmr1*^−/y^ mouse, an increase in intrinsic excitability is also observed [40]. While there are no reported links between EPHB2 and K+ channels, it would be interesting in the future to examine whether this effect is a cell autonomous effect of EPHB2 in cortical neurons or whether it is a compensatory circuit adaptation attempting to normalize cortico-striatal circuit function. Indeed, several studies suggest that increased intrinsic excitability can result from homeostatic mechanisms [41], and the increase we observe in M1 layer V neurons might be the consequence of an initial synaptic hypofunction occurring within the developing and/or mature motor cortical circuitry. Future studies using conditional *EphB2* mutant mice will be helpful to explore these cell autonomous- and temporal-related EPHB2 hypofunction questions.

In summary, we show that a functionally damaging mutation in one *EphB2* allele in female mice is sufficient to produce behavioral phenotypes with potential relevance to ASD, ADHD, and ID and increased excitability of motor cortex pyramidal neurons. While not proving that severe functionally damaging mutations in human *EPHB2* are sufficient to cause ASD or other NDDs, our findings support the notion that full EPHB2 function is critical, particularly in females, for typical expression of behaviors and neurological functions altered in these common NDDs and add to the growing literature that genes involved in synapse development and brain wiring play a disproportionate role in conferring NDD risk.

## Funding and disclosure

This project was supported by a SFARI research grant #240332 from the Simons Foundation (to CWC), NIH grant R01 MH111464 (to CWC), the MUSC Proteogenomics Facility (NIH GM103499), and the MUSC Mouse Behavior Phenotyping Core. The authors declare no competing interests.

## Supplementary information

Supplemental material

## References

[1] Association AP. Diagnostic and Statistical Manual of Mental Disorders, Fifth Edition. Washington, DC, USA: American Psychiatric Association; 2013.

[2] Maenner MJ, Shaw KA, Baio J, EdS, Washington A, Patrick M (2020). Prevalence of Autism Spectrum Disorder Among Children Aged 8 Years - Autism and Developmental Disabilities Monitoring Network, 11 Sites, United States, 2016. MMWR Surveill Summ..

[3] Modabbernia A, Velthorst E, Reichenberg A (2017). Environmental risk factors for autism: an evidence-based review of systematic reviews and meta-analyses. Mol Autism..

[4] Vorstman JAS, Parr JR, Moreno-De-Luca D, Anney RJL, Nurnberger JI, Hallmayer JF (2017). Autism genetics: opportunities and challenges for clinical translation. Nat Rev Genet..

[5] Lord C, Elsabbagh M, Baird G, Veenstra-Vanderweele J (2018). Autism spectrum disorder. Lancet..

[6] Lord C, Brugha TS, Charman T, Cusack J, Dumas G, Frazier T (2020). Autism spectrum disorder. Nat Rev Dis Primers..

[7] Won H, Mah W, Kim E (2013). Autism spectrum disorder causes, mechanisms, and treatments: focus on neuronal synapses. Front Mol Neurosci..

[8] Zhen L, Shao T, Luria V, Li G, Li Z, Xu Y (2018). EphB2 Deficiency Induces Depression-Like Behaviors and Memory Impairment: Involvement of NMDA 2B Receptor Dependent Signaling. Front Pharmacol..

[9] Zhang RX, Han Y, Chen C, Xu LZ, Li JL, Chen N (2016). EphB2 in the Medial Prefrontal Cortex Regulates Vulnerability to Stress. Neuropsychopharmacology..

[10] Zhang Z, Ye M, Li Q, You Y, Yu H, Ma Y (2019). The Schizophrenia Susceptibility Gene OPCML Regulates Spine Maturation and Cognitive Behaviors through Eph-Cofilin Signaling. Cell Rep..

[11] Attwood BK, Bourgognon JM, Patel S, Mucha M, Schiavon E, Skrzypiec AE (2011). Neuropsin cleaves EphB2 in the amygdala to control anxiety. Nature..

[12] Yoshikawa A, Nishimura F, Inai A, Eriguchi Y, Nishioka M, Takaya A (2018). Novel rare variations in genes that regulate developmental change in N-methyl-d-aspartate receptor in patients with schizophrenia. Hum Genome Var..

[13] Sanders SJ, Murtha MT, Gupta AR, Murdoch JD, Raubeson MJ, Willsey AJ (2012). De novo mutations revealed by whole-exome sequencing are strongly associated with autism. Nature..

[14] Sloniowski S, Ethell IM (2012). Looking forward to EphB signaling in synapses. Semin Cell Dev Biol..

[15] Zisch AH, Kalo MS, Chong LD, Pasquale EB (1998). Complex formation between EphB2 and Src requires phosphorylation of tyrosine 611 in the EphB2 juxtamembrane region. Oncogene..

[16] Kalo MS, Pasquale EB (1999). Multiple in vivo tyrosine phosphorylation sites in EphB receptors. Biochemistry..

[17] Holland SJ, Gale NW, Gish GD, Roth RA, Songyang Z, Cantley LC (1997). Juxtamembrane tyrosine residues couple the Eph family receptor EphB2/Nuk to specific SH2 domain proteins in neuronal cells. EMBO J..

[18] Henkemeyer M, Orioli D, Henderson JT, Saxton TM, Roder J, Pawson T (1996). Nuk controls pathfinding of commissural axons in the mammalian central nervous system. Cell..

[19] Sahin M, Greer PL, Lin MZ, Poucher H, Eberhart J, Schmidt S (2005). Eph-dependent tyrosine phosphorylation of ephexin1 modulates growth cone collapse. Neuron..

[20] Fischbach GD, Lord C (2010). The Simons Simplex Collection: a resource for identification of autism genetic risk factors. Neuron..

[21] Kong A, Frigge ML, Masson G, Besenbacher S, Sulem P, Magnusson G (2012). Rate of de novo mutations and the importance of father’s age to disease risk. Nature..

[22] Kohl S, Heekeren K, Klosterkotter J, Kuhn J (2013). Prepulse inhibition in psychiatric disorders–apart from schizophrenia. J Psychiatr Res..

[23] Kuo HY, Liu FC. Synaptic Wiring of Corticostriatal Circuits in Basal Ganglia: Insights into the Pathogenesis of Neuropsychiatric Disorders. eNeuro. 2019;6. 10.1523/ENEURO.0076-19.2019 PMC6553570 31097624

[24] Robichaux MA, Chenaux G, Ho HY, Soskis MJ, Dravis C, Kwan KY (2014). EphB receptor forward signaling regulates area-specific reciprocal thalamic and cortical axon pathfinding. Proc Natl Acad Sci U S A..

[25] Robichaux MA, Chenaux G, Ho HY, Soskis MJ, Greenberg ME, Henkemeyer M (2016). EphB1 and EphB2 intracellular domains regulate the formation of the corpus callosum and anterior commissure. Dev Neurobiol..

[26] Buxbaum JD (2009). Multiple rare variants in the etiology of autism spectrum disorders. Dialogues Clin Neurosci..

[27] McEwen BS, Milner TA (2017). Understanding the broad influence of sex hormones and sex differences in the brain. J Neurosci Res..

[28] Yuhas J, Cordeiro L, Tassone F, Ballinger E, Schneider A, Long JM (2011). Brief report: Sensorimotor gating in idiopathic autism and autism associated with fragile X syndrome. J Autism Dev Disord..

[29] Dines M, Grinberg S, Vassiliev M, Ram A, Tamir T, Lamprecht R (2015). The roles of Eph receptors in contextual fear conditioning memory formation. Neurobiol Learn Mem..

[30] Talebian A, Henkemeyer M (2019). EphB2 receptor cell-autonomous forward signaling mediates auditory memory recall and learning-driven spinogenesis. Commun Biol..

[31] Alapin JM, Dines M, Vassiliev M, Tamir T, Ram A, Locke C (2018). Activation of EphB2 Forward Signaling Enhances Memory Consolidation. Cell Rep..

[32] Grunwald IC, Korte M, Wolfer D, Wilkinson GA, Unsicker K, Lipp HP (2001). Kinase-independent requirement of EphB2 receptors in hippocampal synaptic plasticity. Neuron..

[33] Henderson JT, Georgiou J, Jia Z, Robertson J, Elowe S, Roder JC (2001). The receptor tyrosine kinase EphB2 regulates NMDA-dependent synaptic function. Neuron..

[34] Contractor A, Rogers C, Maron C, Henkemeyer M, Swanson GT, Heinemann SF (2002). Trans-synaptic Eph receptor-ephrin signaling in hippocampal mossy fiber LTP. Science..

[35] Henkemeyer M, Itkis OS, Ngo M, Hickmott PW, Ethell IM (2003). Multiple EphB receptor tyrosine kinases shape dendritic spines in the hippocampus. J Cell Biol..

[36] Hoogenraad CC, Milstein AD, Ethell IM, Henkemeyer M, Sheng M (2005). GRIP1 controls dendrite morphogenesis by regulating EphB receptor trafficking. Nat Neurosci..

[37] Zhu XN, Liu XD, Zhuang H, Henkemeyer M, Yang JY, Xu NJ (2016). Amygdala EphB2 Signaling Regulates Glutamatergic Neuron Maturation and Innate Fear. J Neurosci..

[38] Valenti M, Pino MC, Mazza M, Panzarino G, Di Paolantonio C, Verrotti A. Abnormal Structural and Functional Connectivity of the Corpus Callosum in Autism Spectrum Disorders: a Review. Review Journal of Autism and Developmental Disorders. 2020;7:46–62.

[39] Marco EJ, Hinkley LB, Hill SS, Nagarajan SS (2011). Sensory processing in autism: a review of neurophysiologic findings. Pediatr Res..

[40] Zhang Y, Bonnan A, Bony G, Ferezou I, Pietropaolo S, Ginger M (2014). Dendritic channelopathies contribute to neocortical and sensory hyperexcitability in Fmr1(-/y) mice. Nat Neurosci..

[41] Pratt KG, Aizenman CD (2007). Homeostatic regulation of intrinsic excitability and synaptic transmission in a developing visual circuit. J Neurosci..

