# Supplementary material for: Sex-dependent role for EPHB2 in brain development and autism-associated behavior
Source: Neuropsychopharmacology. 2021 Mar 1;46(11):2021–9. doi: 10.1038/s41386-021-00986-8 (PMC8429442; doi:10.1038/s41386-021-00986-8)
Supplement: Supplementary file 1 — Supplemental material [file 41386_2021_986_MOESM1_ESM.pdf]

SUPPLEMENTARY MATERIALS AND METHODS

Site-directed mutagenesis. The 21 different variants in *EPHB2* were generated via site-directed mutagenesis. First, a polymerase chain reaction (PCR) was performed using a pCDNA3 plasmid expressing the coding sequence of the human *EPHB2* transcript (NM_001309193.2) and primers that contain each mutation. The PCR mix and the PCR program are described in detail in supplemental Figure 1. Template DNA was digested using Dpn1 (NEB#R0176S) according to manufacturer specification. The DNA PCR product was purified using a PCR purification kit (Qiagen #28104), following the kit protocol, and was eluted with 30µL of ultrapure water. Competent bacteria (Fisher #18-265-017) were transformed using purified DNA and plated on LB agar plates containing ampicillin (Fisher # 17-125-425GM). Finally, individual bacteria colonies were selected and incubated in 3mL of liquid LB containing ampicillin at 37°C under agitation overnight. The plasmids were purified from bacteria using a Miniprep kit (Qiagen #27104) following the kit protocol and sent for sequencing using 6 pairs of primers that span the entire *EPHB2* sequence, to confirm the presence of the correct mutations and the accuracy of the rest of the sequence. The sequences of the primers used to sequence the plasmids are described in supplemental Figure 1.

HEK293T cell transfection. HEK293T cells (ATCC #ATCC® CRL-3216™) were cultured in 12-well plates at 350,000 cells/well in 1mL/well of Dulbecco's modified Eagle medium (DMEM, Fisher # 11-960-044) supplemented with 10% fetal bovine serum (Fisher # 16-000-044), 1% penicillin-streptomycin (Sigma # P0781), and 1% L-glutamine (Sigma # G7513), and placed at 37°C/5% CO_2 in a humidified incubator. To overexpress EPHB2 mutant proteins, ~24 hours after plating, HEK293T cells were transfected with the pCDNA3 plasmids expressing the different *EPHB2* variants using the calcium phosphate transfection method. Briefly, 500ng of *EPHB2* plasmid, 300ng of GFP (Addgene), $CaCl_2$ (250mM final), and HBS (1X final) were combined and 100µl of the mixture was added to each well of a plate containing the HEK293T cells. Finally, 24 hours after transfection, HEK293T cells expressing the different *EPHB2* mutants were harvested. Culture medium was removed, cells were rinsed with ice-cold 1X PBS, and 200µL of 2X SAB with 10% BME (Fisher # 21-010-046) was added to the cells. The cells were harvested using a cell scraper and the cell lysate was passed a few times through a 25G needle to disrupt genomic DNA. Cell lysates were boiled at 98°C for 5-10 minutes, centrifuged at 15,000g for 10 min, and the supernatant was collected and stored at -20°C.

Western Blot. Samples were boiled at 98°C for 5 minutes, and 2µL of the HEK293T cell lysate containing the overexpressed EPHB2 mutant proteins were resolved by SDS-PAGE using 10% gels (BIO-RAD #4568034). The proteins were transferred to PVDF membranes (BIO-RAD #1704157) using a rapid transfer system (BIO-RAD, Trans-Blot® Turbo™ Transfer System). Membranes were then incubated for 1 hour in Odyssey blocking solution (LI-COR #927-40000; supplemented with 0.1% Tween, 0.01% SDS, 0.02% azide) at room temperature under agitation, in primary antibodies (rabbit anti-HA, 1:1000, Sigma #H6908; rabbit anti-phospho EPH ([1], 1:1000) diluted in Odyssey blocking solution (supplemented with 0.1% Tween and 0.02% azide) overnight at 4°C under agitation, and in secondary antibodies (LI-COR #926-32211; Goat anti-rabbit, 1:20000) diluted in Odyssey blocking solution (supplemented with 0.1% Tween, 0.01% SDS, 0.02% azide) for 1 hour and 30 minutes at room temperature under agitation. Finally, membranes were imaged using the Odyssey CLx Western blot system (LI-COR).

Behavior Testing: Social interaction. Mice were allowed to explore a three-arena apparatus (Stoelting #60450) for 10 minutes. Mice were then removed while a non-familiar conspecific mouse (same age and sex) and a novel object were placed in holding chambers in the side arenas. An interaction zone (circle with a 16.5cm diameter) was defined around each holding chamber. Experimental mice were returned to the center arena and recorded for 10 minutes using ANY-maze behavior tracking software (Stoelting) to measure the time spent interacting with the novel mouse and novel object. Data are reported as time spent in each interaction zone. ***Pup ultrasonic vocalization (USV)***. Pups separated from their mother generate distress USVs. For identification purposes, long-lasting subcutaneous tattoos (Ketchum) were performed on postnatal day 5/6 (P5/6) pups. USVs were recorded from isolated pups at P5/6 and P10 for 3 minutes immediately following separation from their mother and littermates, in a sound-attenuated chamber, using Avisoft UltraSoundGate equipment (UltraSoundGate 116Hb with Condenser Microphone CM16; Avisoft Bioacoustics, Germany). USVs were quantified using Avisoft SASLab Pro (Avisoft Bioacoustics, German). Data are reported as number of USVs generated during the 3 minutes of recording. ***Elevated Plus Maze***. Mice were placed in the center of an elevated plus maze that has 2 open arms and 2 closed arms (Stoelting #60140) in white light (100 lux) and recorded for 5 minutes using ANY-maze behavior tracking software (Stoelting). Data are reported as time spent in open arms. ***Open Field***. Mice were placed in a white open field box ($44cm^2$) in white light (130 lux) and recorded for 5 minutes using ANY-maze behavior tracking software (Stoelting). Data are reported as time spent in a predefined zone (14cm by 14cm) in the well-lit center of the box. ***Locomotor activity.*** Mice were placed in the dark for 1 hour in an Open Field

Activity, Infrared Photobeam Activity Test Chamber (Med Associates), that has 2 arrays of photobeams to measure vertical (jumping, rearing) and horizontal locomotor movements. Data are reported as total distance traveled, jump time, and vertical and horizontal repetitive fine movement time. ***Fear conditioning***. During fear conditioning (FC), the mice learn to associate an aversive experience with a specific context and with a specific cue (auditory tone). After 2 minutes of acclimation in a fear conditioning chamber (Med Associates), mice were exposed three times (once/minute) to an auditory tone (30 seconds; 90 dB) followed each time by a mild footshock (0.5mA). Freezing behavior was recorded using a videotracking system (Video Freeze V2.6; Med Associates). Twenty four hours later, the freezing behavior of mice was recorded when the mice returned to the chamber (Test - context) for 6 minutes, and when the mice were re-exposed to the auditory tone in a new context for another 6 minutes (3 minutes without tone (Test – altered context) followed by 3 minutes during which the tone was played (Test – cue/altered context)). Data are reported as percent of time the mouse is immobile. ***Startle response to shock, acoustic startle response, and prepulse inhibition***. Mice were placed in Plexiglas and wire grid animal holders (Med Associates #ENV-264C) attached to a load cell platform (Med Associates #PHM-250) contained within a sound-attenuated chamber. To measure startle response to shock, mice were exposed to foot shocks delivered by S/A Aversive Stimulators (Med Associates #ENV-414S) connected to the wire grid floors of the animal holder. To measure acoustic startle response, mice were exposed to 5 white-noise pulses/intensity (38ms) at 4 different intensities (70, 80, 90, and 100 dB) in a randomized order with variable inter-trial intervals (10-20s). Startle responses were measured using the Startle Reflex System and Advanced Startle software program (Med Associates). Displacements of the load cell stabilimeter were converted into arbitrary units by an analog-to-digital converter interfaced to a personal computer. To measure prepulse inhibition, acoustic startle responses were measured from mice exposed to white-noise at 100 dB, preceded by silence and preceded by a prepulse (that does not elicit startle responses) at 70 dB and at different frequencies (4, 12 and 20Hz). In WT animals, exposure to a prepulse stimulus attenuates/inhibits the startle response to white noise at 100 dB. The percentage of prepulse inhibition was calculated as following: 100 – (startle response at 100 dB preceded by a prepulse/startle response at 100 dB preceded by silence x 100).

Myelin staining. 2-3 month-old *EphB2*$^{+/-}$ and WT mice were anesthetized with Ketamine/Xylazine diluted in 0.9% saline (120mg/kg and 16mg/kg, respectively), and perfused transcardially with 4% paraformaldehyde (PFA). The brains were post-fixed overnight in 4% PFA, cryoprotected in 30% sucrose and sectioned at 40µm using a cryotome (Leica ITEM). Sections were stained for myelin

using the BrainStain Imaging kit (ThermoFisher #B34650; FluoroMyelin, 1:300), following the kit protocol, and imaged using an epifluorescence microscope. Cortical thickness, corpus callosum thickness, and dorsal striatal area were measured using ImageJ software.

Electrophysiology. All acute-slice electrophysiological experiments were performed in *EphB2*$^{+/-}$ mice and their WT littermates. Animals were live-decapitated, and acute coronal slices (300μm thickness), containing M1 motor cortex, were obtained using a slicer (Leica VT1200s) in ice-cold artificial cerebrospinal fluid (ACSF) containing (in mM): 127 NaCl, 2.5 KCl, 1.2 NaH_2PO_4, 24 $NaHCO_3$, 11 D-glucose, 1.2 $MgCl_2$, and 2.40 $CaCl_2$, 0.4 Na-ascorbate (pH 7.4, 315-320 mOsm). Kynurenic acid (5 mM) was added to ACSF to avoid overactivity of glutamatergic receptors during slicing. Slices were then transferred to normal ACSF (without kynurenic acid) to recover at 37°C for 30 minutes and then room temperature ACSF for an additional 30-minute recovery period prior to recording. All solutions were continually equilibrated with 95% O_2 and 5% CO_2. Layer V (LV) pyramidal neurons of M1 were visualized with infrared differential interference contrast optics (DIC/infrared optics) and identified by their location, apical dendrites, and spiking patterns in response to depolarizing current injection. Unless stated otherwise, all electrophysiological experiments were performed in whole cell voltage clamp mode at -70 mV using borosilicate pipettes (4-6 MΩ) pulled using a NARISHIGE puller (NARISHIGE, PG10) from borosilicate tubing (Sutter Instruments) and filled by an internal solution containing (in mM): 140 $CsMetSO_4$, 5 KCl, 1 $MgCl_2$, 0.2 EGTA, 11 HEPES, 2 NaATP, 0.2 Na_2GTP and 0.1 $CaCl_2$, pH 7.2–7.4 (pH 7.2**,** 290–295 mOsm). All recording data were acquired and analyzed by an amplifier AXOPATCH 200B (Axon Instruments), digitizer BNC2090 (X National instruments) and software AxoGraph v.1.7.0, Clampfit v 8.0 (pClamp, Molecular devices) and MiniAnalysis Program v.6.0.9 (Synaptosoft). Data were filtered at 2 kHz by the AXOPATCH 200B amplifier (Axon Instruments) and digitized at 10-20 kHz via AxoGraph v.1.7.0. software. ***Evoked postsynaptic currents*.** The evoked postsynaptic responses of M1 pyramidal neurons in LV were elicited by field stimulation of excitatory afferents in LV at a frequency of 0.05 Hz (0.05 c^{-1}) - 3 stimuli per second. The low-intensity pulses of stimulated current (25–100 μA, 50–100 μs duration) were applied through a fine-tipped (~2-3 μm), bipolar stimulating electrode made from borosilicate theta glass capillary tubing (Warner Instruments). AMPA-receptor-mediated excitatory postsynaptic currents (EPSCs) were recorded at -70 mV (reversal potential for GABA current) to minimize $GABA_A$ current. Inhibitory postsynaptic currents (IPSCs) mediated by $GABA_A$ receptors were recorded at 0 mV (reversal potential for AMPA currents) to eliminate the current through AMPA-receptors. To calculate the AMPA/GABA ratio, the amplitude of the AMPA response recorded at -70 mV was

divided by the amplitude of the GABA response at 0 mV. ***Action potentials.*** Action potentials were initiated by a series of depolarizing current pulses of increasing intensities (0-1000 pA in 50 pA steps; 1 s duration at 0.2 Hz) and recorded in whole cell current clamp mode. The holding transmembrane current was clamped at -70 mV. The pipette solution was a potassium- based internal solution (in mM): 145 KGlu, 5 KCl, 1 $MgCl_2$, 0.2 EGTA, 11 HEPES, 2 NaATP, 0.2 Na_2GTP and 0.1 $CaCl_2$, pH 7.2–7.4 (pH 7.2, 290–295 mOsm). Intrinsic excitability is presented as a graph of AP number vs depolarization level at each pulse. ***NMDA currents and AMPA/NMDA ratio.*** Evoked postsynaptic responses of pyramidal cells in layer V of M1 cortex (coronal slices) were elicited by field stimulation of excitatory afferents at a frequency of 0.05 Hz (0.05 c^{-1}). Low-intensity pulses of stimulated current (200 µs duration) were applied through a fine-tipped (~2-3 µm), bipolar stimulating electrode made from borosilicate theta glass capillary tubing (Warner Instruments). To standardize fiber stimulation, stimulated current within a 150–200 µA range, corresponding to 150-200% of threshold intensity stimulation (typically ~100 µA). AMPA and NMDA receptor-mediated excitatory postsynaptic currents (AMPA- and NMDA-responses) were recorded respectively at -70 and +50 mV of membrane potential. Recordings were made in the presence of picrotoxin (50 µM) to block inhibitory postsynaptic currents mediated by GABA-A receptors. The amplitude of AMPA responses was calculated at the maximum current value, and the amplitude of the NMDA response was calculated from the value at 50 ms post-stimulation. These values were used to calculate the AMPA/NMDA ratio.

RT-qPCR. Postnatal day 2-6 or ~P60 WT and *EphB2*$^{+/-}$ mice were live-decapitated, the cortex was dissected, flash-frozen in dry ice, and stored at -80°C. RNA extraction was performed using the miRNeasy Mini kit (Qiagen #1038703), following the protocol kit. Total RNA was reverse-transcribed using Superscript III (Invitrogen) with random hexamers, following the protocol kit. Quantitative real-time PCR was performed using the CFX96 qPCR instrument (Bio-Rad), the iTaq Universal SYBR Green Supermix (Bio-Rad), and primers specific to *EphB2* (Forward: 5'CAACGGTGTGATCCTGGACTAC3', Reverse: 5'CACCTGGAAGACATAGATGGCG3' used for Suppl. figure 4A and B; Forward: 5'GATGGTACATCCCCCATCAG3', Reverse: 5'GCCAGTTGTTCTGGCTTGAC3' used for Suppl. figure 4C and D). GAPDH was used to normalize gene expression in each sample. The data are reported as delta delta Ct.

1. Sahin, M., et al., *Eph-dependent tyrosine phosphorylation of ephexin1 modulates growth cone collapse.* Neuron, 2005. **46**(2): p. 191-204.

SUPPLEMENTAL FIGURES AND TABLES

A PCR mix

Reagent	Volume	Reference
5X phusion polymerase buffer	5uL	NEB #M0530S
Plasmid at 50ng/uL	1uL	
Forward primer at 25ng/uL	5uL	
Reverse primer at 25ng/uL	5uL	
dNTPs	2uL	Fisher #N8080260
Phusion DNA polymerase	1uL	NEB #M0530S
Ultrapure water	32uL	

B PCR program

	Temperature	Time
	95C	30 sec
16 X	95C	30 sec
16 X	51C	1 min
16 X	68C	17 min
	72C	5 min
	4C	∞

C Primers' sequences for *EphB2* sequencing

Primer	Sequence
Primer 1 Forward	5' ATGAAGTTCTCGGTGCGTGA 3'
Primer 2 Forward	5' GAGGTTGTCCATCAGGAACC 3'
Primer 3 Forward	5' GTGATCCTGGACTACGAGCT 3'
Primer 4 Forward	5' GATCTTTGTAGCCATCAAGA 3'
Primer 5 Forward	5' CAATCAAGACGTAATCAACG 3'
Primer 6 Reverse	5' CAGTGTTGATTTTCATGACG 3'

Supplemental Figure 1: Materials and methods information.
A, B. PCR mix (A) and PCR program (B) for site-directed mutagenesis.
C. Primers' sequences for EphB2 sequencing.

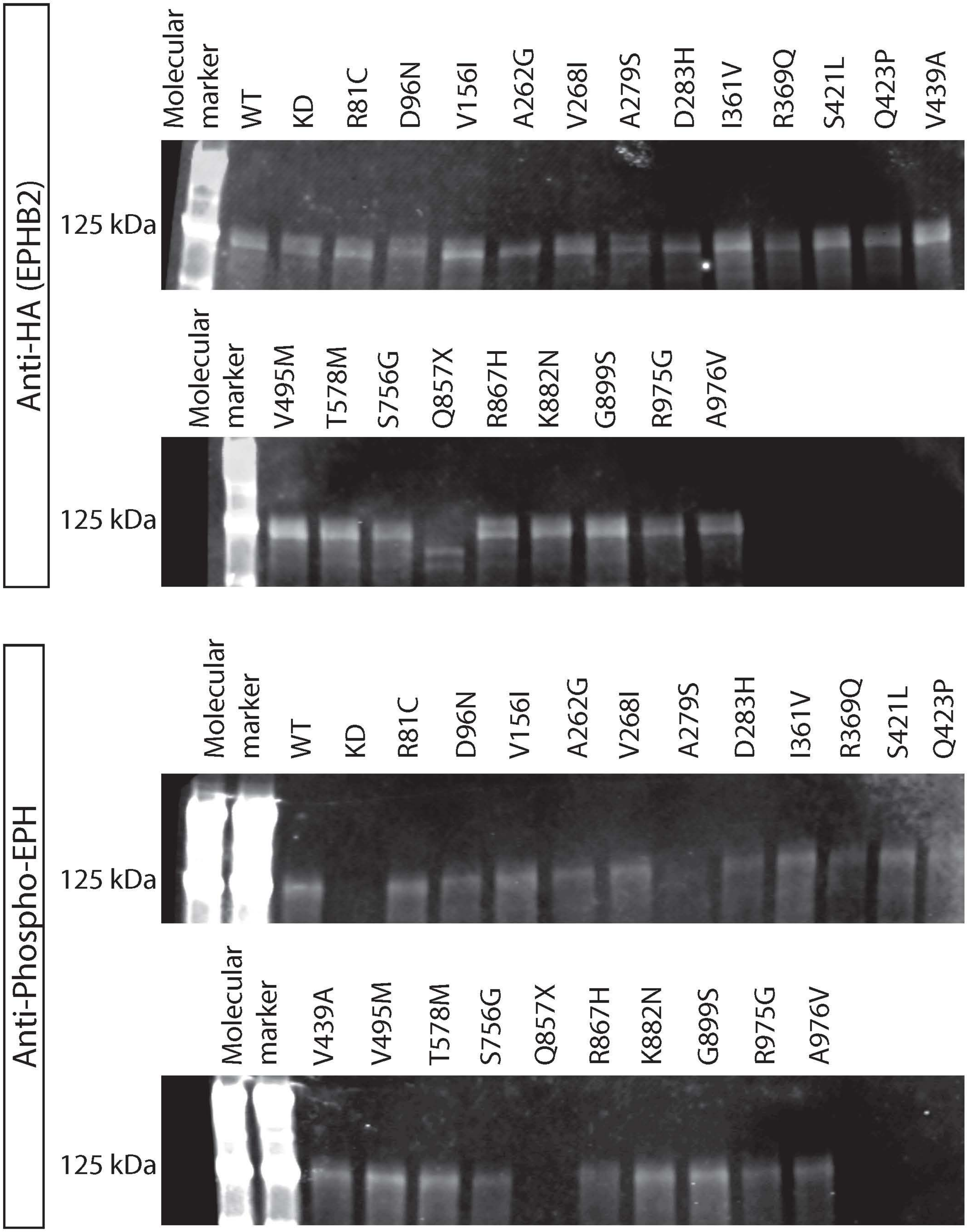

Supplemental Figure 2: Western blots of HA-EPHB2 and of Y602 phospho-EPH for all EPHB2 variants. WT: wild-type; KD: kinase-dead.

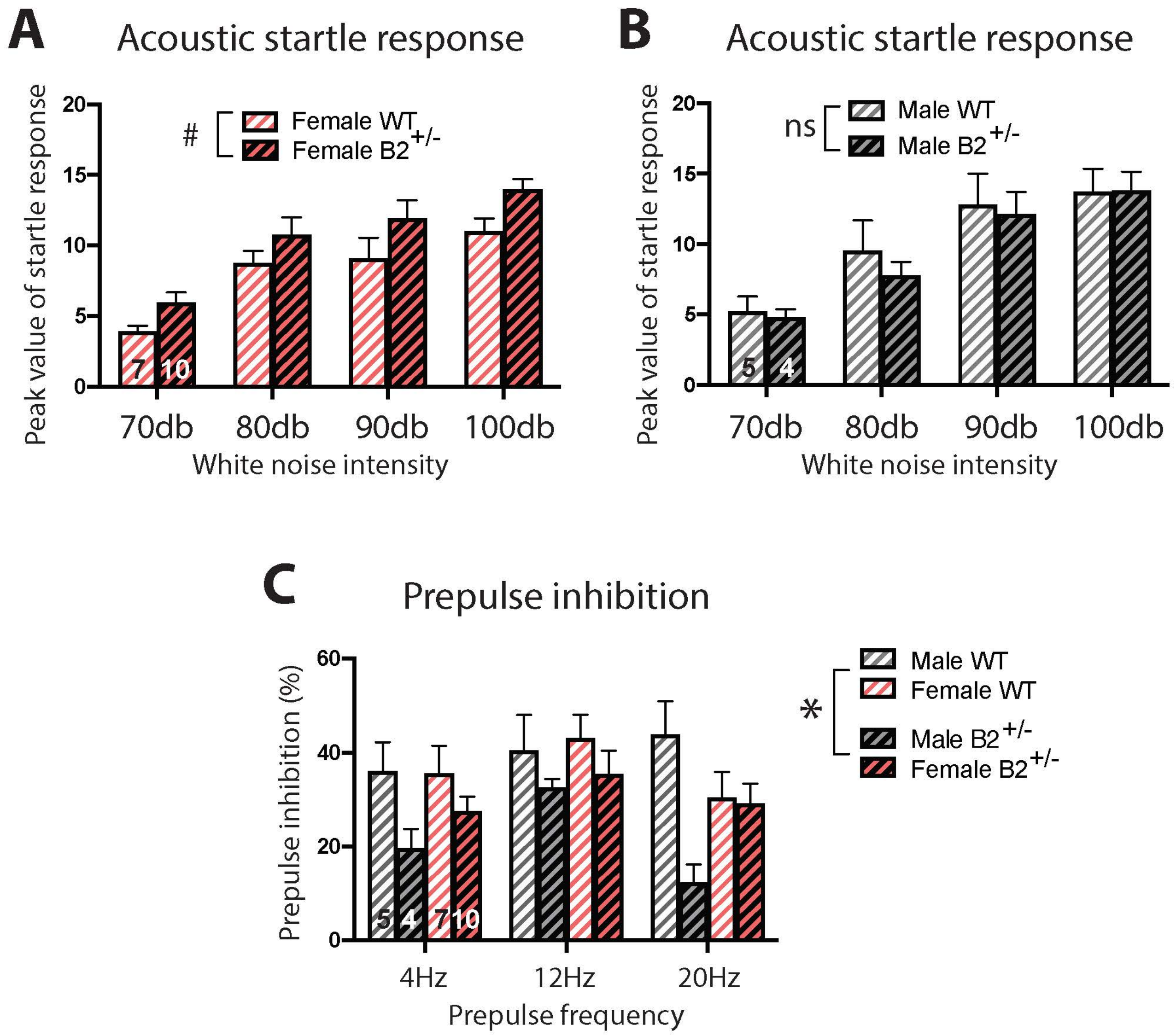

Supplemental Figure 3: Sensory characterization of EphB2+/- mice.
A, B. EphB2+/- females, but not males, display a trend to increased acoustic startle response (two-way ANOVA; for females, statistical trend to main effect of genotype: $p=0.0713$). **C.** EphB2+/- mice present a deficit in prepulse inhibition (three-way ANOVA; main effect of genotype: $p=0.0249$). Data are represented as mean ± SEM. #$p<0.1$, *$p<0.05$, ns: not significant. The number of animals is indicated winthin each bar for each experiment.

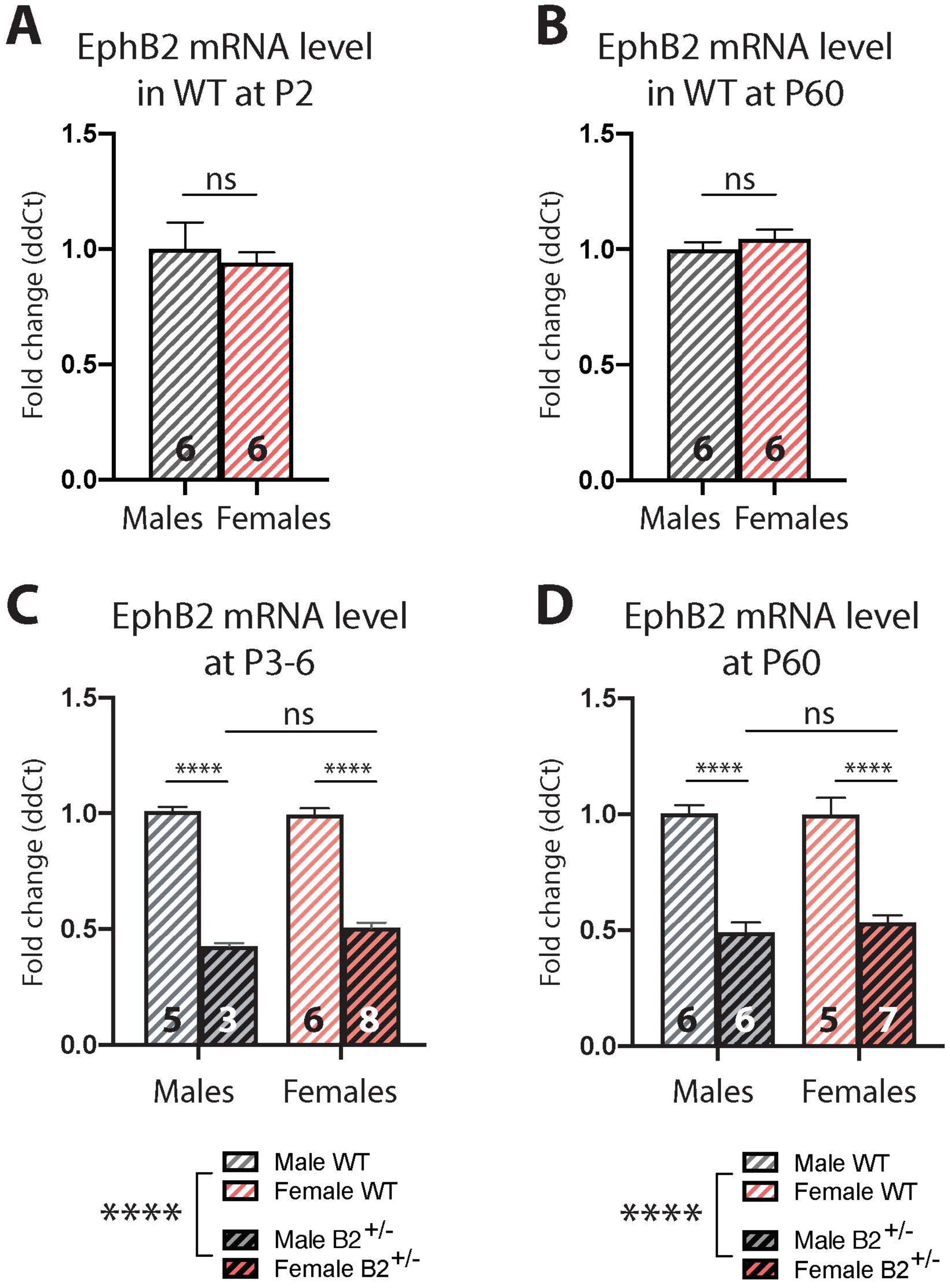

Supplemental Figure 4: No difference in EphB2 mRNA level of expression between males and females.
A, B. qPCR analysis from the whole cortex at P2 (A) and at two months old (B) shows no difference in EphB2 expression levels between WT males and females (t-test). **C, D.** qPCR analysis at P3-6 from the whole brain (C) and at two months old from the whole cortex (D) shows a ~50% decrease in EphB2 expression levels between EphB2+/- and WT mice (two-way ANOVA; main effect of genotype: $p<0.0001$), but no difference in EphB2 expression levels between EphB2+/- males and females. Since the sequence deletion in one allele of the EphB2 gene in the EphB2+/- mouse model leads to a truncated mRNA that gives rise to a null protein (frameshift), the forward primer was designed to recognize the deleted sequence in EphB2 mRNA and the reverse primer was designed to recognize a sequence outside of the deleted sequence. This design allows for the specific quantification of the WT EphB2 mRNA giving rise to a normal protein in both WT and EphB2+/- mice. Data are represented as mean ± SEM. Number of animals is indicated within each bar for each experiment. ****$p<0.0001$, ns: not significant.

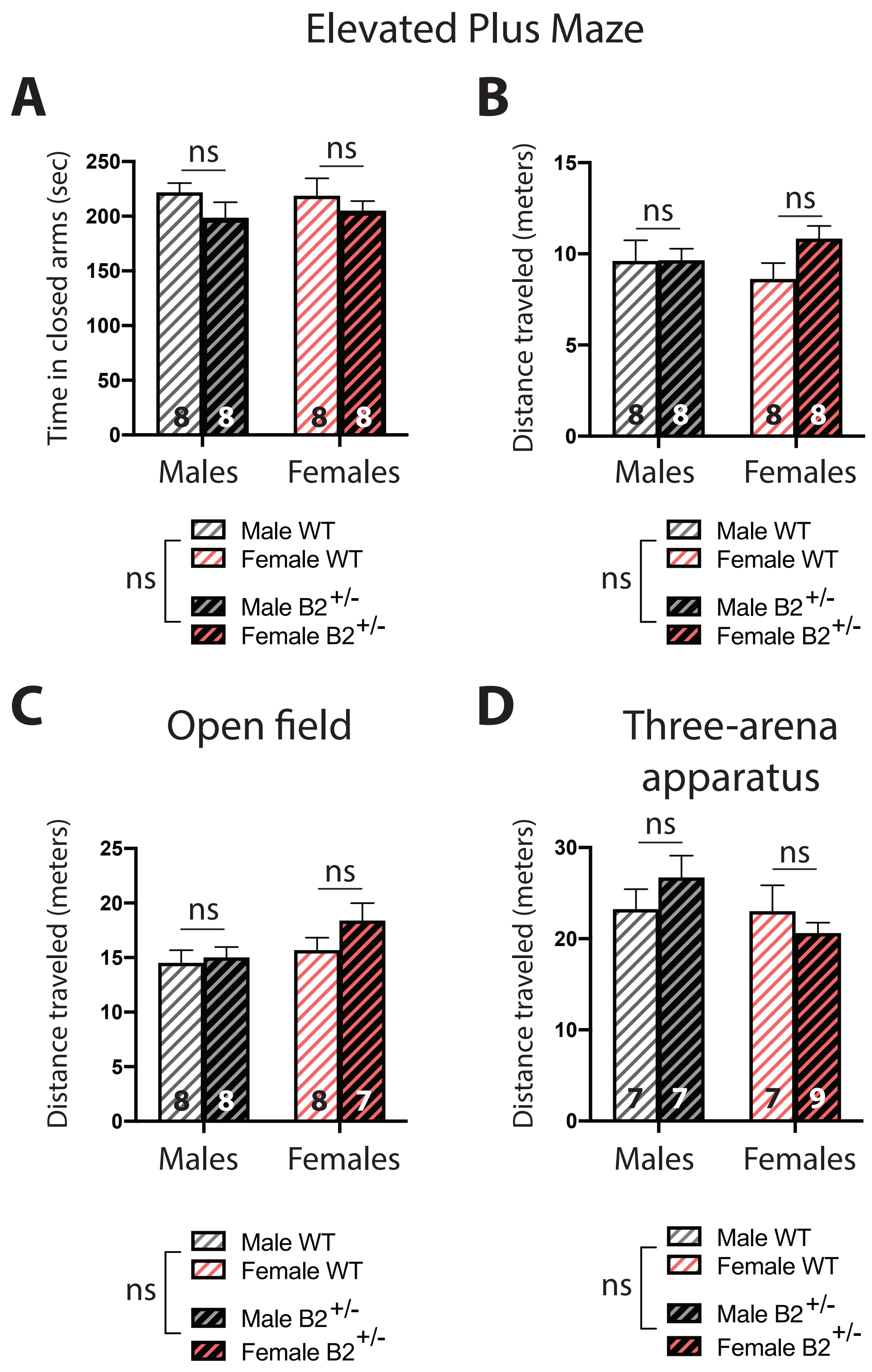

Supplemental Figure 5: A, B, C, D. No difference in the time spent in the closed arms in the elevated plus maze (A), or in the distance traveled in the elevated plus maze (B), in the open field (C), and in the three-arena apparatus during social interaction (D) between WT and EphB2+/- mice (two-way ANOVAs). Data are represented as mean ± SEM. Number of animals is indicated within each bar for each experiment. ns: not significant.

Supplemental table 1: p-values for t-tests

Assay	p-value
Elevated Plus Maze	0.1487
Open field	0.9158
Corpus callosum thickness	0.4517
Cortex thickness	0.8832
Striatum area	0.9860
E/I ratio	0.6479
EphB2 mRNA level at P2	0.6379
EphB2 mRNA level in adults	0.4100

Supplemental table 2: p-values for two-way ANOVAs

Assay	Interaction	Genotype	Sex	Other variable
Elevated Plus Maze	0.3698	0.1332	0.0607	N/A
Open field	0.5641	0.9457	0.6433	N/A
Corpus callosum thickness	0.5710	0.3915	0.3786	N/A
Cortex thickness	0.9230	0.9230	0.4111	N/A
Striatum area	0.6967	0.8844	0.7699	N/A
E/I ratio	0.9643	0.6464	0.6959	N/A
Intrinsic excitability	<0.0001	0.0334	N/A	Current <0.0001
Social interaction	0.7658	0.4606	N/A	Interaction zone 0.0002
Pup USV	0.0301	0.3515 Post-hoc at P5/6: 0.0540		Age 0.6133
Repetitive rearing	0.3865	0.0234 Post-hoc for females: 0.0540 Post-hoc for males: 0.4986	0.1401	N/A
Horizontal repetitive movements	0.0251	0.2642 Post-hoc for females: 0.0385 Post-hoc for males: 0.6315	0.0007	N/A
Jumping	0.1173	0.3337	0.0181	N/A
Locomotor activity	0.1167	0.0011 Post-hoc for females: 0.0018 Post-hoc for males: 0.3034	0.1861	N/A
Test - Altered context/cue	0.0420	0.007 Post-hoc for females: 0.0026 Post-hoc for males: 0.8275	0.4673	N/A
Test - Context	0.3159	0.3233	0.0378	N/A
Startle response to footshock	0.5467	0.8410	0.3611	N/A
Training - Context	0.9717	0.6111	0.0215	N/A
Training - Cue	0.2832	0.0437 Post-hoc for females: 0.0616 Post-hoc for males: 0.7272	0.0629	N/A
Test - Altered context	0.5313	0.2230	0.2383	N/A
Acoustic startle response Males	0.8682	0.7294		White noise <0.0001
Acoustic startle response Females	0.8425	0.0713		White noise <0.0001
Prepulse inhibition	0.6174	0.0494	N/A	Frequency 0.0008

Supplemental table 3: p-values for three-way ANOVAs

Assay	Interaction	Genotype	Sex	Third variable
Social interaction	0.7339	0.5446	0.0144	Interaction zone 0.0002
Pup USV	0.8362	0.4953	0.9273	Age 0.9884
Prepulse inhibition	0.0036	0.0249	0.6009	Frequency 0.0003
